# Perioperative Transcutaneous Electrical Acupoint Stimulation Reduces Postoperative Pain in Patients Undergoing Thoracoscopic Surgery: A Randomized Controlled Trial

**DOI:** 10.1155/2024/5365456

**Published:** 2024-06-30

**Authors:** Jianming Liu, Keqin Zhang, Yongyan Zhang, Feng Ji, Haifeng Shi, Yi Lou, Hua Xu

**Affiliations:** ^1^ Department of Anesthesiology Yueyang Hospital of Integrated Traditional Chinese and Western Medicine Shanghai University of Traditional Chinese Medicine, Shanghai 200437, China; ^2^ Department of Anesthesiology Pulmonary Hospital Tongji University, Shanghai 200433, China

## Abstract

**Objectives:**

This study aimed to determine the effects of perioperative transcutaneous electrical acupoint stimulation (TEAS) on postoperative pain management in patients undergoing thoracic surgery.

**Methods:**

In the prospective, randomized, controlled study, a total of 84 patients undergoing video-assisted thoracoscopic surgery (VATS) were randomly allocated to the TEAS group (Group T) or control group (Group C). Patients in the Group T received TEAS at Neiguan (PC6) and Hegu (LI4) acupoints for 30 min before anesthesia induction and 30 min after thoracoscopic surgery. Patients in the Group C received the same placement of electrodes but without electrical stimulation. The numeric rating scale (NRS) pain score, remifentanil consumption, demand for rescue analgesics and incidence of postoperative nausea and vomiting (PONV), patient satisfaction, and the levels of plasma *β*-endorphin (EP) and IL-6 were recorded.

**Results:**

Patients in the Group T had significantly lower NRS pain scores at 6 h, 12 h, 24 h, and 48 h after surgery than those in the Group C. Compared with Group C, patients in Group T had lower remifentanil consumption during operation, lower demand for rescue analgesics and lower rate of PONV within 24 h after surgery. Patients in Group T also had lower IL-6 content, higher *β*-EP content and higher satisfaction degree than those in the Group C.

**Conclusions:**

Perioperative TEAS significantly decreased postoperative pain and rescued analgesia requirements and the incidence of PONV in patients undergoing thoracoscopic surgery, with a higher patient satisfaction. This trial is registered with ChiCTR2100051841.

## 1. Introduction

With the improvement of medical services level, the conventional thoracotomy has been gradually replaced by video-assisted thoracoscopic surgery (VATS) for resectable lung cancer [1]. Despite of the widespread of the VATS, persistent postoperative pain is still a challenge for anesthesiologists and surgeons, which causes both physical discomfort and daily dysfunction. Previous study reported that the incidence of postoperative pain after VATS ranged from 20% to 47% [2], and poorly controlled postoperative pain after VATS has been associated with chronic postsurgical pain [3]. In addition, persistent and severe postthoracotomy pain can negatively affect postoperative recovery and increase the risk of pulmonary complications, such as atelectasis and pneumonia [4, 5].

Current treatments for persistent pain after VATS include analgesia drugs, antidepressants, regional nerve block, and psychotherapy [6]; however, no cure has been guaranteed. Previous studies have shown that acute postoperative pain was an important risk factor for chronic pain after surgery and acute postoperative pain could transform into chronic pain [7–9]. Therefore, perioperative pain management may reduce the occurrence of chronic pain after surgery. Transcutaneous electrical acupoint stimulation (TEAS) has been shown to reduce the consumption of opioids during general anesthesia [10] and was used to reduce the incidence of acute postsurgical pain [11]. Therefore, the present study hypothesized that TEAS might be a suitable method to reduce postoperative analgesia in patients undergoing VATS pulmonary resection.

Although TEAS can effectively alleviate acute postoperative pain, the mechanisms underlying this analgesic action of TEAS have not yet been fully elucidated. A previous study has found that electroacupuncture can alleviate acute pain through inflammation signaling pathway [12]. In addition, several studies reported that electroacupuncture relieved pain by increasing the expression of *β*-endorphin (*β*-EP) [13, 14]. Thus, in the present study, we also explore the effects of TEAS on the levels of plasma IL-6 and *β*-EP in patients undergoing VATS.

## 2. Materials and Methods

### 2.1. Trial Design

This study was a prospective, randomized, controlled trial. Patients received elective VATS in the Department of Anesthesiology of Yueyang Hospital of Integrated Traditional Chinese and Western Medicine between March 2021 and March 2022 were recruited. The trial was registered at Chinese Clinical Trial Registry (ChiCTR2100051841). The study was approved by the Shanghai Yueyang Hospital Ethics Committee (Shanghai, China) (2021-083), and written informed consents were obtained from all participants.

### 2.2. Participants

This study included consecutive patients aged 18–75 years, with American Society of Anesthesiology (ASA) physical status classification of I–III, who were scheduled for elective VATS pulmonary resection including lobectomy, segmentectomy, and wedge resection under general anesthesia. All patients had a body mass index (BMI) 18–28 kg/m^2^ and received postoperative patient control intravenous analgesia (PCIA). Patients with contraindications to TEAS (scar or skin damage at the stimulation site and electronic devices such as pacemakers or other implanted medical electronic devices in the body), unable to communicate, long-term use of analgesics (including nonsteroidal anti-inflammatory drugs and opioids), receiving bilateral VATS, history of chest surgery, history of chronic pain or neuropathic pain, autoimmune diseases, and persistent infection were excluded.

### 2.3. Randomization and Blinding

Participants were randomized into Group C and Group T (TEAS at bilateral LI4 and PC6) in 1 : 1 ratio using computer-generated random number tables. Group allocation was typed on separate pages, folded, and concealed in sequentially numbered sealed opaque envelopes by an anesthesia nurse. Outcomes were assessed by trained researchers who were masked to the treatment allocation. The patients and acupuncturists who involved with the intervention were not blinded to the allocation. The allocation was unknown among other clinical staff, including surgeons, anesthesiologists, and ward staff.

### 2.4. Study Intervention

**Figure 1:**
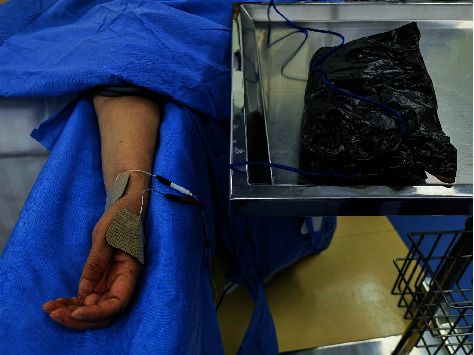
Location of acupoints.

The qualified acupuncturists who were not involved in the anesthesia or follow-up conducted TEAS in accord with recommendations from the Standards for Reporting of Controlled Trials in Acupuncture [15]. In short, electrodes were attached to the skin surface of the acupoint and connected to the Han's acupoint nerve stimulator by leads (HANS200A, Nanjing Jisheng Medical Appliance Co., Ltd, Nanjing, China). Patients in the Group T received TEAS at bilateral PC6 point (Neiguan, a key acupoint of the hand-jueyin pericardium meridian) located between the tendons of the flexor carpi radialis and palmaris longus muscles, one-sixth of the distance between the distal transverse wrist crease and the antecubital crease, along the course of the median nerve and LI4 point (Hegu, a key acupoint of the hand-yangming large intestine meridian) on the back of the hand, between the first and second metacarpal bones, and at the midpoint of the radial side of the second metacarpal bone (Figure 1). The device provided “disperse-dense” waves with frequencies of 2/200 Hz and continuous stimulation for 30 min before anesthesia induction and for 30 min after surgery, respectively. The “De Qi” sensations of heaviness, numbness, and swelling at the point of stimulation were determined as the maximal tolerance of the stimulation intensity. After 30 min of TEAS, anesthesia induction was initiated. After surgery, TEAS was administrated for 30 min in the postanesthetic care unit (PACU). Patients received electrode attachment without stimulation in the Group C. The nerve stimulator was placed in a black that the surgical team and anesthesiologists were blinded for group assignment.

### 2.5. Blood Sample Collection and Measurements

Blood samples were collected in anticoagulant tubes before TEAS (T_0_), discharge from PACU (T_1_), and 24 h after surgery (T_2_). When phlebotomy was performed, we tried our best to minimizing hemolysis or platelet activation. Blood samples were collected into anticoagulant tubes and immediately centrifuged at 4000 r/min for 10 min, and the plasma was collected and stored in a −80°C freezer. The levels of *β*-EP and IL-6 in the plasma were measured using a high-sensitivity enzyme-linked immunosorbent assay kit (ZCIBIO Technology Co., Ltd, Shanghai, China).

### 2.6. Outcomes

#### 2.6.1. Primary Outcome

The primary outcome of the study was postoperative pain intensity, which was evaluated using the Numerical Rating Scale (NRS). Pain intensity was assessed at 6 h, 12 h, 24 h, 48 h, 72 h, 1 month, 2 months, and 3 months postoperatively. Area under the curve (AUC), a valid statistical method was adopted to combine the pain scores across the 6–72 h for comparison. The area under the curve (AUC) of the NRS scores was calculated by multiplying the time interval with the NRS scores using GraphPad Prism V.7 (GraphPad Software, San Diego, CA, USA).

#### 2.6.2. Secondary Outcomes

The secondary outcomes include remifentanil consumption during general anesthesia, PONV, and demand for rescue analgesics by 24 h postoperatively. The Visual Analogue Scale (VAS) was used to evaluate patient satisfaction at 72 h postoperatively. Besides, we measured the levels of *β*-EP and IL-6 in plasma of patients at T_0_, T_1_, and T_2_.

### 2.7. Procedures

Anesthesia was induced through target-controlled infusion (TCI) with plasma concentrations of propofol 3 *μ*g/ml and remifentanil 4 ng/ml and cis-atracurium 0.2 mg/kg intravenously. The position of double-lumen endobronchial tube was confirmed by fiberoptic bronchoscopy after intubation. Continuous inhalation of 1% sevoflurane and TCI with propofol and remifentanil were titrated to maintain bispectral index (BIS) between 4–60 and stable hemodynamics. If necessary, additional cis-atracurium and sufentanil were administered. After endotracheal extubation, patients were transferred to the PACU. All patients received patient-controlled analgesia (PCA) after surgery: 100 *μ*g sufentanil and 50 mg flurbiprofen axetil was diluted to 100 ml with 0.9% saline, and a self-administered dose of 2 ml was programmed with a 20 min lockout period and no background infusion. Parecoxib sodium 40 mg was administered as rescue analgesia for patients whose NRS pain score ≥4 after surgery.

### 2.8. Data Collection

Specially trained research personnel collected all data. For pain evaluation, we used the 11-point numeric rating scale (NRS), where an NRS score of 0 represented “no pain” and a score of 10 represented “worst pain imaginable”. Each patient received verbal and written explanations of the scale. The outcome assessors came to the ward and followed up the patients face to face to assess pain intensity at 6 h, 12 h, 24 h, 48 h, and 72 h after surgery. At 1 month, 2 months, and 3 months after surgery, the patients were followed up using a telephonic questionnaire. Remifentanil consumption during general anesthesia was recorded. The incidence of PONV and demand for rescue analgesics were recorded up to 24 h postoperatively. Patient satisfaction score was obtained at 72 h postoperatively. The levels of *β*-EP and IL-6 in the plasma were measured.

### 2.9. Statistical Analysis

The sample size was calculated using Pass software version 15.0 (NCSS, Kaysville, UT, USA). Based on the results of our pilot trial, the score of NRS in the control group at 6 h after surgery was 4.21 ± 1.58 while in the TEAS group was 3.31 ± 1.21, with an equal randomization and assuming a loss to follow-up of 10%; a sample size of 41 patients in each group will ensure that the power was at least 80% to detect the difference at a significant level of 0.05 using a two-sided *t*-test. All statistical analysis was performed by SPSS 23.0. Student's *t*-test was used to compare continuous variables in normally distributed data described as the mean ± standard deviation (SD), while the Mann–Whitney *U* test was used to compare continuous variables in non-normal distribution data described as medians (quartiles). Categorical variables represented as frequency and percentages were compared using Pearson's *χ*^2^ test or Fisher's exact test. In addition, a two-way repeated-measures analysis of variance using a Bonferroni correction for multiple comparisons was used to evaluate the level of biomarkers in plasma between the two groups. *P* < 0.05 was considered statistically significant.

## 3. Results

### 3.1. Preoperative Patient Characteristics

**Figure 2:**
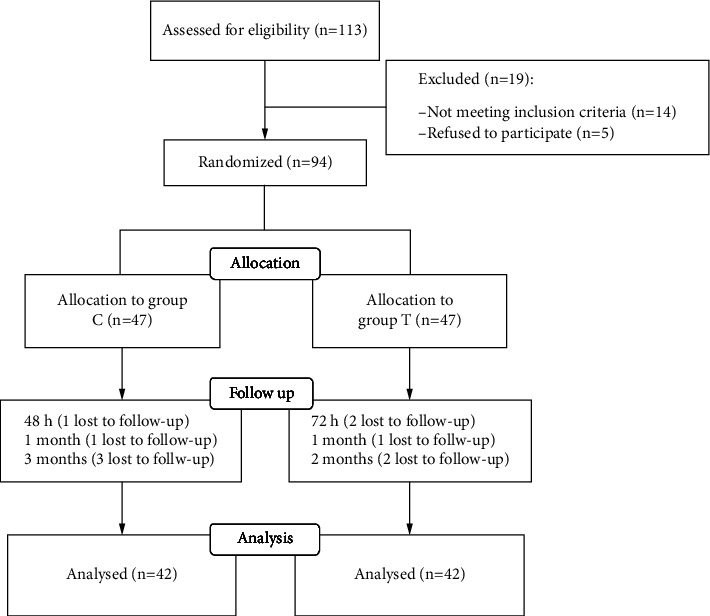
Flowchart of participants in the trial.

We assessed 113 patients for eligibility to participate in this study. Among them, 14 patients did not meet the inclusion criteria and 5 declined to participate. The remaining 94 patients were enrolled in the study. Later, 5 patients from the Group C and 5 patients from the Group T were lost during the follow-up. The final statistics included 84 patients, 42 in Group C and 42 in Group T. The participant flow diagram is shown in Figure 2. There were no significant differences in demographic characteristics and anesthetic and surgical parameters between the two groups (Table 1).

### 3.2. Primary Outcome

#### 3.2.1. Postoperative Pain Intensity

The Group T had a significantly lower NRS pain score at 6 h, 12 h, 24 h, and 48 h after surgery compared with the Group C (*P* < 0.01) (Table 2). However, there was no significant difference in NRS pain score at 72 h, 1 month, 2 months, and 3 months postoperatively between the two groups (Table 2). The AUC of pain in the Group T was significantly lower than that in the Group C at 6–72 h after surgery (mean ± SD, 268.7 ± 56.1 vs. 324.8 ± 66.7, *P* < 0.01) (Table 2). In brief, perioperative TEAS was superior in analgesia during the first 2 days after surgery.

#### 3.2.2. Secondary Outcomes

Remifentanil consumption during operation was significantly higher in the Group C than in the Group T (mean ± SD, 1.50 ± 0.51 vs. 1.23 ± 0.35, *P* < 0.01) (Table 1). Compared with the Group C, the frequency of rescue analgesia at 24 h after surgery in the Group T were significantly lower (*n* [%], 36 [85.7%] vs. 25 [59.5%], *P* < 0.01) (Table 3).

The incidence of PONV at 24 h after surgery was 35.7% in the Group C, which was significantly higher than 14.3% in the Group T (*P*=0.023) (Table 3). The satisfaction score of patients on analgesia at 72 h after surgery in the Group T was higher than that in the Group C (mean ± SD, 5.95 ± 1.82 vs. 3.66 ± 1.58, *P* < 0.01) (Table 4).

**Figure 3:**
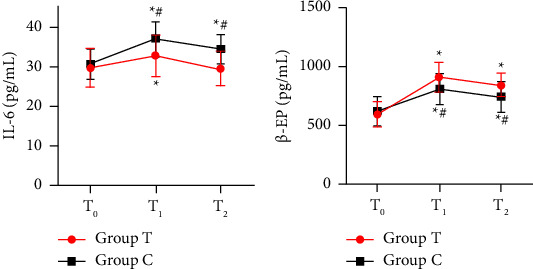
Plasma levels of IL-6 and *β*-EP in the two groups. (a) Plasma levels of IL-6 between the two groups. (b) Plasma levels of *β*-EP between the two groups. Values are presented as the mean ± standard (SD). Group T, transcutaneous electrical acupoint stimulation group; Group C, control group. T_0_, before transcutaneous electrical acupoint stimulation; T_1_, discharge from postanesthetic care unit; T_2_, 24 h after surgery; IL, interleukin; EP, endorphin. ^*∗*^*P* < 0.05 vs. T_0_. ^#^*P* < 0.05 vs. Group T.

The levels of IL-6 at T_1_ and T_2_ were significantly lower in the Group T than that in the Group C (*P* < 0.05). Besides, compared with the Group C, the levels of *β*-EP at T_1_ and T_2_ were significantly higher in the Group T (*P* < 0.05). The levels of IL-6 and *β*-EP at T_1_, T_2_ were significantly higher compared with the baseline level in the Group C (*P* < 0.05). The level of IL-6 at T_1_ was significantly higher compared with the baseline level in the Group T (*P* < 0.05). There was no significant difference in the level of IL-6 at T_2_ compared with the baseline level in the Group T. The levels of *β*-EP at T_1_ and T_2_ were significantly higher compared with the baseline level in the Group T (*P* < 0.05). (Figure 3).

## 4. Discussion

The present study demonstrated that perioperative TEAS can significantly reduce postoperative pain, remifentanil consumption, and the incidence of PONV and improve satisfaction scores in patients undergoing VATS. In addition, we also found that perioperative TEAS was associated with high *β*-EP and low IL-6 in patients who receiving VATS. The present study provided an effective preventive approach to persistent pain after VATS and a potential mechanism of TEAS in inhibiting the pain intensity after thoracoscopic surgery.

Persistent pain after thoracic surgery can lead to respiratory complications, such as hypoxia and pulmonary infection, and negatively affect postoperative recovery [4]. Poorly controlled acute pain after VATS also leads to chronic postsurgical pain, which can severely impair physical function and quality of life. Although there are some treatments for postoperative pain, such as paravertebral nerve block, thoracic epidural block, and intercostal nerve block, the postoperative pain in patients undergoing VATS remains at a high incidence (ranging from 59% to 90%) [8, 16]. As traditional Chinese acupuncture therapy, TEAS is a noninvasive form of acupuncture interventions which applies electrical stimulation onto acupuncture points using transcutaneous electrodes. Previous studies have reported that TEAS is successfully used in the reduction of postoperative pain and is well received by patients without adverse effects [17, 18]. However, the impacts of TEAS on postoperative pain in patients undergoing VATS and its underlying mechanism are still unclear. In the present study, we found that administering TEAS during the perioperative period can significantly reduce the rest NRS pain score at 6 h, 12 h, 24 h, and 48 h after surgery. However, we did not observe any difference between groups in NRS scores from 3 days to 3 months after surgery. Furthermore, perioperative TEAS can effectively reduce the demand for rescue analgesic, which also indicated that TEAS had an analgesic effect on postoperative acute pain. Our results seem to indicate that perioperative TEAS has no role in relieving chronic pain. This is different from the results of previous studies. A multicenter, prospective, randomized, controlled trial revealed that TEAS at combined acupoints before anesthesia induction in patients undergoing mastectomy is associated with a decreased incidence of chronic pain at 6 months after surgery compared with the sham intervention [18]. This may be due to differences in the selected acupoints. There is no consensus on the number of acupoints that should be selected in acupuncture therapy. In real-world practice, acupuncturists decide the number of acupoints based on the theory of traditional Chinese medicine and their personal experiences. In the abovementioned study, PC6 and RN17 were selected for combined acupoint stimulation [18]. PC6 and LI4 were selected for combined acupoint stimulation in the present study.

Remifentanil is a selective and ultrashort-acting synthetic opioid, which is commonly used in general anesthesia and pain management. Nonetheless, remifentanil can lead to a wide range of side effects including bradycardia, hypotension, respiratory depression, muscle rigidity, opioid-induced tolerance, and hyperalgesia [19]. Previous studies have demonstrated that perioperative TEAS intervention can reduce the intraoperative opioid consumptions and improved postoperative recovery in patients undergoing nephrectomy and patients received sinusotomy [10, 19]. Consistent with previous studies, we also observed a significantly reduction in intraoperative remifentanil consumption in the TEAS group than in the control group, suggesting that perioperative TEAS may exert an analgesic effect. Nausea and vomiting are common gastrointestinal symptoms following opioid administration; they are two key stumbling blocks that affect the recovery of patients [20]. The results of our research also showed that the incidence of PONV at 24 h after VATS in Group T was lower than that in Group C, which may be because TEAS has a good analgesic effect on patients undergoing VATS surgery, decreases opioids use, and thus reduces the incidence of PONV.

Surgical injury and incision can activate the immune system and cause inflammation [21]. Previous evidence has found that the mechanisms of TEAS analgesia involve several systems including endorphin system and immunomodulation system [22]. *β*-EP is an endogenous analgesic substance released by the pituitary chiefly, which can inhibit pain and reduce stress level [23]. IL-6 is an important proinflammatory cytokine that causes inflammation and stress. In this study, we found that perioperative TEAS increased the plasma levels of *β*-EP and decreased the contents of IL-6 in patients undergoing VATS. Moreover, the concentration of interleukin-6 decreased to preoperative levels at 24 hours after surgery in Group T. These results suggest that perioperative TEAS has the distinct advantage of inhibiting inflammatory outbreaks. These data indicated that TEAS not only has a potential analgesic effect on postoperative pain but also helps regulate inflammation response and stress levels.

Endogenous opioids evoked by electroacupuncture are frequency dependent and have been proven to play a central role in the pain inhibition [24]. Previous studies found that 2/100 Hz (dense-and-disperse, DD) mode of stimulation was able to induce simultaneous release of three opioid peptides to produce synergistic analgesic effects [25, 26]. Therefore, we followed the stimulation protocol described in previous studies. PC6 and LI4 are important acupoints for analgesia in meridian theory which can increase pain threshold, alleviate microvascular spasms, and influence the frontal cortex to participate in the pain modulation of ventrolateral thalamic nucleus. Previous studies have reported that LI4 and PC6 had an important role in pain relief in different parts of the body including incision in chest, which were acupuncture points commonly used for analgesia [27–29]. In addition, stimulation of the acupuncture point PC6 was also used for preventing PONV [30].

The current research also has some unavoidable limitations. First, perioperative TEAS could not be administered in a patient-blinded and acupuncturists-blinded manner, which may have introduced bias to some extent in outcome monitoring. However, the outcome assessors were unaware of the group assignments. The stimulator was placed in a black box so that both the surgeon and the anesthesiologist were blinded. We chose sham stimulation on the same acupoints as the control group instead of nonacupoints stimulation as positive placebo in the present study. Future studies could add a set of nonacupoints stimulation to minimize bias. Second, long-term changes of plasma *β*-EP and IL-6 after surgery failed to be measured. Finally, multicenter clinical large samples and randomized clinical trials need to be done in the future.

## 5. Conclusion

Perioperative TEAS significantly decreased postoperative pain and rescued analgesia requirements and the incidence of PONV in patients undergoing thoracoscopic surgery, with a higher patient satisfaction. These results provided new insights into the potential benefits of perioperative TEAS for postoperative pain management after VATS and supported its use in clinical practice.

## Figures and Tables

**Table 1:** Demographic and surgical characteristics in the two groups.

Characteristics	Group C (*n* = 42)	Group T (*n* = 42)	*P*
Male	17 (40.5)	24 (57.1)	0.127
Age (years)	54.90 ± 11.06	58.17 ± 7.97	0.125
BMI (kg/m^2^)	22.79 ± 2.48	23.62 ± 2.26	0.114
ASA status (I/II/III)	17/22/3	19/22/1	0.64
Smoking before operation	13 (31)	13 (31)	1
Surgical site (left/right)	15/27	16/26	0.821
Operation type			0.162
Lobectomy	23 (54.8)	29 (69)	
Segmentectomy	7 (16.7)	8 (19)	
Wedge resection	12 (28.6)	5 (11.9)	
Duration of surgery (min)	88.07 ± 43.15	86.38 ± 39.61	0.852
Fluid input during surgery			
Crystalloid input (ml)	300 (250–400)	265 (250–475)	0.877
Colloid input (ml)	500 (500–600)	500 (500–600)	0.549
Blood loss (ml)	50 (20–50)	50 (21–50)	0.922
Urine (ml)	100 (0–200)	200 (100–200)	0.064
Remifentanil consumption (mg)	1.50 ± 0.51	1.23 ± 0.35	0.006

Values are presented as the mean ± SD, *n* (%) or median (interquartile range). BMI, body mass index; ASA, American Society of Anesthesiologists; Group C, control group; Group T, transcutaneous electrical acupoint stimulation group.

**Table 2:** Postoperative NRS pain scores at different times.

	Group C	Group T	*P*
NRS AUC at 6–72 h after surgery	324.8 ± 66.7	268.7 ± 56.1	<0.01
6 h after surgery	5.0 (4.0–7.0)	4.0 (4.0-5.0)	<0.01
12 h after surgery	6.0 (5.0–7.0)	4.5 (3.0–6.0)	<0.01
24 h after surgery	5.0 (3.0–6.0)	4.0 (3.0–5.0)	<0.01
48 h after surgery	5.0 (4.0–6.0)	4.0 (3.0–5.0)	<0.01
72 h after surgery	4.0 (3.0–5.0)	4.0 (3.0–5.0)	0.308
1 month after surgery	3.0 (3.0–4.0)	3.0 (2.0–3.0)	0.577
2 months after surgery	2.0 (2.0–3.0)	2.0 (2.0–2.0)	0.665
3 months after surgery	2.0 (2.0–3.0)	2.0 (1.0–2.0)	0.484

Values are presented as the mean ± SD or median (interquartile range). NRS, numeric rating scale; AUC, area under the curve; Group C, control group; Group T, transcutaneous electrical acupoint stimulation group.

**Table 3:** Rescue analgesia and incidence of PONV.

	Group C (*n* = 42)	Group T (*n* = 42)	*P*
Rescue analgesia by 24 h after surgery	36 (85.7%)	25 (59.5%)	<0.01
PONV by 24 h after surgery	15 (35.7%)	6 (14.3%)	0.023

Values are presented as *n* (%). PONV, postoperative nausea and vomiting; Group C, control group; Group T, transcutaneous electrical acupoint stimulation group.

**Table 4:** Satisfaction of patients.

	Group C	Group T	*P*
The satisfaction score of patients	3.66 ± 1.58	5.95 ± 1.82	<0.01

Values are presented as the mean ± SD. Group C, control group; Group T, transcutaneous electrical acupoint stimulation group.

## Data Availability

The data used to support the findings of this study are available on request from the corresponding author. The data are not publicly available due to privacy or ethical restrictions.
